# Prognostic model based on disulfidptosis-related lncRNAs for predicting survival and therapeutic response in bladder cancer

**DOI:** 10.3389/fimmu.2024.1512203

**Published:** 2024-12-02

**Authors:** Lirui Han, Hankai Yang, Xuan Jiang, Ziyu Zhou, Chang Ge, Kairan Yu, Guofang Li, Wei Wang, Yubo Liu

**Affiliations:** ^1^ Department of Life and Pharmaceutical Sciences, School of Chemical Engineering, Ocean and Life Sciences, Dalian University of Technology, Panjin, China; ^2^ Ministry of Education (MOE) Key Laboratory of Bio-Intelligent Manufacturing, Dalian University of Technology, Dalian, China; ^3^ Department of Thoracic Surgery, Liaoning Cancer Hospital & Institute, Cancer Hospital of Dalian University of Technology, Liaoning, Shenyang, China

**Keywords:** disulfidptosis, bladder cancer, long non-coding RNA, machine learning, prognosis

## Abstract

**Background:**

With poor treatment outcomes and prognosis, bladder cancer remains a focus for clinical research in the precision oncology era. However, the potential of disulfidptosis, a novel cell death mechanism, and its related long non-coding RNAs to support selective cancer cell killing in this disease is still unclear.

**Methods:**

We identified key disulfidptosis-related lncRNAs in bladder cancer, constructed a prognostic risk model with potential therapeutic targets, and confirmed the findings through quantitative PCR analysis.

**Results:**

We identified five crucial lncRNAs (*AC005840.4*, *AC010331.1, AL021707.6*, *MIR4435-2HG* and *ARHGAP5-AS1*) and integrated them into a predictive model centered on disulfidptosis-associated lncRNAs. Reliability and validity tests demonstrated that the lncRNA prediction index associated with disulfidptosis effectively discerns patients’ prognosis outcomes. Additionally, high-risk patients exhibited elevated expression levels of genes involved in the PI3K-Akt signaling pathway, extracellular matrix organization, and immune escape mechanisms, which are associated with poor prognosis. Notably, high-risk patients demonstrated higher sensitivity to *Sorafenib*, *Oxaliplatin* and *MK-2206*, underscoring the promise of these lncRNAs as precise therapeutic targets in bladder cancer.

**Conclusion:**

By revealing the predictive importance of disulfidptosis-associated lncRNAs in bladder cancer, our research offers new perspectives and pinpoints potential therapeutic targets in clinical environments.

## 1 Introduction

Bladder cancer (BC) is one of the most common malignant tumors affecting the urinary system, posing substantial challenges to global healthcare (1, 2). The incidence of BC varies widely, encompassing both occult, non-invasive types and highly aggressive forms with high mortality rates. The clinical course of BC is highly variable; some cases progress slowly and are prone to recurrence, necessitating long-term invasive monitoring (3, 4). In contrast, other cases exhibit aggressive behavior, spreading to surrounding tissues and distant organs, resulting in poor prognosis and reduced survival rates. Effective treatment of BC requires a personalized, multidisciplinary approach, including surgery, chemotherapy, radiotherapy, and immunotherapy (5, 6). Despite the diversity and advancements in treatment options, the heterogeneity and varied clinical presentations of BC present significant challenges for treatment and prognosis. Identifying novel biomarkers is crucial for selecting optimal chemotherapy regimens, determining responsive patient groups, achieving early diagnosis, and enhancing the efficacy of diagnostic and therapeutic interventions (7–9). In recent years, long non-coding RNAs (lncRNAs) have garnered attention for their selective expression in tumor cells, making them potential markers of cancer. Numerous studies have demonstrated that lncRNAs, screened from cancers such as breast and colon cancer, show strong potential in predicting tumor classification, treatment response, and prognosis (10), making them effective tools for prognostic prediction, tumor microenvironment assessment, and the selection of immunotherapy and chemotherapy (10–13).

Disulfidptosis, a newly identified form of metabolic cell death, is considered a promising avenue for cancer therapy. According to research by Professors Gan Boyi and Chen Junjie, the primary characteristic of disulfidptosis is initiated when cells with elevated *SLC7A11* expression face glucose deprivation. In preclinical studies, glucose transporter (GLUT) inhibitors have been shown to induce disulfidptosis in *SLC7A11*-overexpressing cancer cells, effectively suppressing tumor growth while minimizing toxicity to healthy tissues (14). This research provides new strategies for developing innovative cancer treatments and, due to its distinct mechanism from known cell death types, may offer new pathways for treating cancers resistant to traditional therapies (15, 16). It supports selective killing of cancer cells by targeting cancer metabolism from a precision oncology perspective (17). Additionally, the discovery of disulfidptosis highlights the metabolic vulnerability of *SLC7A11* overexpressing cancer cells (16, 18), offering new insights into cancer treatment by targeting their dependency on glucose and NADPH. Notably, the application of disulfidptosis in cancer treatment requires further research and validation. Existing preclinical studies indicate that tumors with high *SLC7A11* levels exhibit greater sensitivity to GLUT inhibitors, presenting a new direction for cancer therapy. However, the potential for utilizing disulfidptosis-triggered tumor-killing strategies in BC remains unclear, and further investigation is needed to clarify the role of associated lncRNAs as biomarkers for guiding treatment and prognosis.

Accordingly, the objective of this study was to discover and confirm new lncRNA-based prognostic indicators related to disulfidptosis, with the aim of enhancing prognostic forecasting for patients with BC. We established and validated a highly accurate prognostic model, examining differences in cellular functions, signaling pathways, and immune characteristics between high- and low-risk groups. We aimed to create a prognostic nomogram to enhance clinical decision-making and personalize treatment by estimating survival probabilities for BC patients. Our findings highlight the regulatory role of disulfidptosis-related lncRNAs in BC progression, paving the way for future precision therapies targeting these molecular markers.

## 2 Materials and methods

### 2.1 Transcriptomic data collection and analysis

We retrieved publicly available transcriptomic data from the TCGA-BLCA database, which provides RNA expression profiles for 412 BC tumor samples and 19 normal tissue samples. These profiles were systematically combined with corresponding clinical factors, including sex, age, stage, and survival information, using Perl version 5.30.0. To ensure data integrity, samples with incomplete clinical or transcriptomic data were excluded from the analysis.

### 2.2 Identification of DRGs

Building on previous studies, a set of disulfidptosis-related genes (DRGs) was identified. With R version 4.2.0, we created a matrix of expressions for lncRNAs associated with disulfitosis using the ‘BiocManager’ and ‘limma’ packages. To ensure the reliability of the DRL expression matrix, we set strict filtering criteria with |Pearson R| > 0.4, *p* < 0.001.

### 2.3 Development and validation of a prognostic prediction model

**Figure 1:**
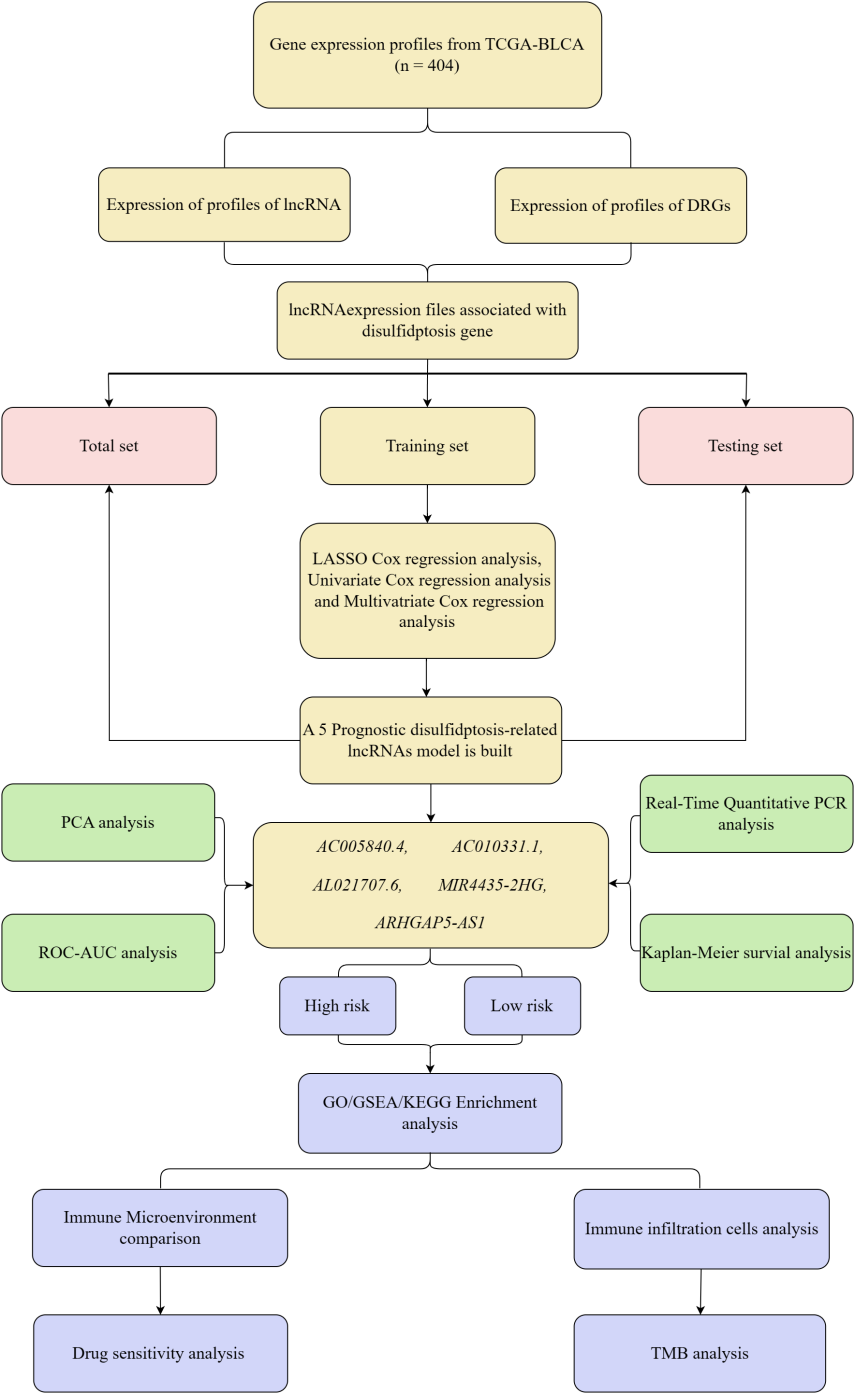
Flow chart of the entire study.

The experimental design for this study is depicted in a schematic flowchart (**Figure 1**). The lncRNA expression matrix was integrated with patient survival information. A cohort of 404 BC patients was split into training and validation groups. The training set underwent LASSO and univariate Cox regression analysis, with key DRLs identified through subsequent multivariate Cox regression (*p* < 0.05). The model’s robustness was confirmed using the validation group and the complete dataset. Five key DRLs were identified, and risk assessments along with heatmaps were generated using R packages. Risk scores were computed using the formula: Risk score = *Σi = lnCoef(i) × Expr(i)*, and patients were subsequently divided into high-risk and low-risk groups based on the median score. Several statistical techniques were employed to evaluate the precision and dependability of the model’s predictions.

### 2.4 Functional enrichment analysis

We utilized enrichment analyses, including GO, KEGG, and GSEA, to investigate the molecular functions and pathways linked to DEGs across various risk levels. Robustness was ensured with *p*-value and q-value thresholds of 0.05, various R packages were used to systematically explore enriched functions and pathways.

### 2.5 Tumor mutational burden and immune escape analysis

Perl scripts were employed to calculate the tumor mutational burden (TMB) for each BC patient. Differential analysis, using advanced software tools, revealed significant variations. To evaluate immune escape mechanisms, The Tumor Immune Dysfunction and Exclusion (TIDE) score was sourced directly out of its repository to evaluate immune escape mechanisms. Immune evasion differences between high- and low-risk BC groups were analyzed using the TIDE score.

### 2.6 Immune characteristic comparison

To assess the immunological variations across different risk levels, we utilized various R packages designed for statistical analysis, presenting the distribution of 22 tumor-infiltrating immune cell types through box plots. Additionally, to evaluate immune cell penetration and checkpoint evaluation, a correlation heatmap was generated using various R packages, including ‘limma,’ ‘tidyverse,’ ‘ggplot2,’ ‘ggpubr,’ ‘ggExtra,’ and ‘reshape2’.

### 2.7 Prediction of drug response and therapeutic outcomes

To assess drug responsiveness and predict therapeutic outcomes in targeted cancer treatments, we utilized the Genomics of Drug Sensitivity in Cancer (GDSC) database. OncoPredict software was employed to estimate patient responses to specific therapies, with patients classified into elevated- and reduced-risk categories.

### 2.8 A real-time quantitative PCR method for RNA extraction and analysis

In our study, RNA was extracted from T24 and SV-HUC-1 cells using Trizol, then transcribed into cDNA with the Sangon Biotech One Step RT-qPCR Kit for subsequent qPCR analysis. The specific primer sequences can be found in **Supplementary Table S1**. Melting curve images are provided in **Supplementary Figure S1** to demonstrate the specificity of lncRNA expression.

## 3 Results

### 3.1 Discovery of key DRLs and prognostic model development in BC

**Figure 2:**
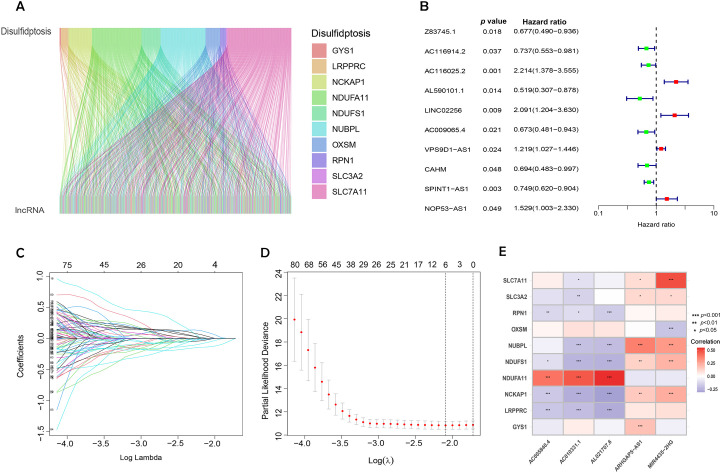
Identification of DRLs and construction of a prognostic DRLs signature in BC. **(A)** Sankey diagram showing the relationship between disulfidptosis genes and disulfidptosis lncRNAs. **(B)** Forest plot of prognostic genes associated with DRLs. **(C)** LASSO coefficients of the DRLs obtained by LASSO analysis. **(D)** Cross-validation of DRLs in LASSO regression. **(E)** Multivariate Cox regression analysis and correlation between bisulfite pendulous genes and DRLs. Statistical significance: *p* < 0.05; *p* < 0.01; *p* < 0.001; ns, no significance.

We extracted transcriptomic data from the TCGA database, identifying 412 sequences expressed in BC and normal tissue samples. Further analysis showed that within the BC lncRNA dataset, disulfidptosis-related genes correlated, satisfying the standards of |Pearson’s R| > 0.4 and *p* < 0.001. Patients with incomplete data were excluded, resulting in a final cohort of 404 BC patients whose lncRNA expression patterns were linked with clinical survival outcomes. These patients were randomly split into two groups: 202 in the training set and 202 in the testing set, with no significant differences in their clinical characteristics. To visualize the connections between DRLs and disulfidptosis-related genes, Sankey diagrams were employed (**Figure 2A**).

Univariate Cox regression, followed by LASSO regression, identified 84 DRLs associated with BC prognosis, underscoring significant predictors of poor outcomes (**Figures 2B–D**). Multivariate Cox regression identified five DRLs significantly linked to overall survival (OS) in TCGA cohorts. The expression patterns of these five DRLs, deemed most influential in determining BC prognosis, including *AC005840.4*, *AC010331.1*, *AL021707.6*, *MIR4435-2HG*, and *ARHGAP5-AS1*, were visualized through heatmaps (**Figure 2E**).

### 3.2 Constructing and validating a prognostic risk assessment mode

**Figure 3:**
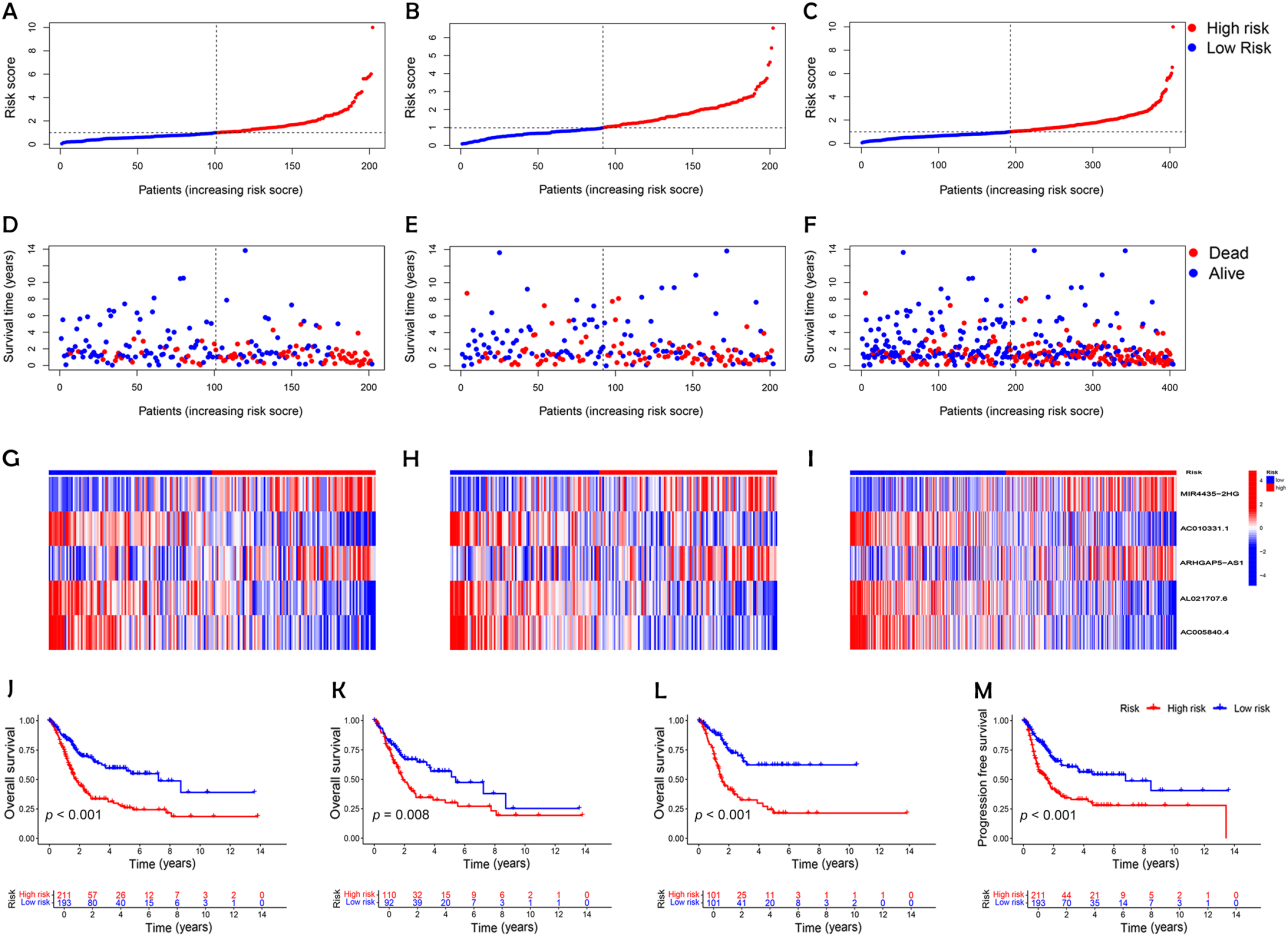
Establishment of a risk prediction model for predicting overall survival of patients with BC. **(A-C)** Distribution of patients in the combined, training, and testing sets according to increasing risk scores (**A**, training set; **B**, testing set; **C**, combined set). **(D-F)** Correlation between survival time and risk scores in three patient groups. (**D**, training set; **E**, testing set; **F**, combined set). **(G-I)** Heatmap of risk scores for five key disulfidptosis-associated lncRNAs across the three groups (**G**, training set; **H**, testing set; **I**, combined set). **(J-L)** Kaplan-Meier survival analysis of OS in the three patient groups (**J**, combined set; **K**, testing set; **L**, training set). **(M)** Progression-free survival analysis of the combined dataset.

Following the identification of key DRLs, formula was developed to determine individual risk levels and produce risk curves. As depicted in **Figures 3A–C**, patients were ordered from low to high risk along the horizontal axis, with the vertical axis displaying their respective risk scores. Based on the median risk score obtained from the training group, the 404 BC patients were categorized into high- and low-risk groups. A survival analysis was performed on all data sets, including the training and testing sets (**Figures 3D–F**). Higher risk scores were associated with increased mortality, with high-risk patients showing shorter survival times (indicated by red circles) and low-risk patients experiencing longer survival (indicated by blue circles). In the test cohort, low-risk patients demonstrated a markedly higher survival rate than those at high risk (*p* < 0.05; **Figure 3K**). Kaplan-Meier survival curves demonstrated that OS was significantly greater in patients with lower risk than in those classified as higher risk (*p* < 0.001; **Figures 3J, L**). These results emphasize the model’s effectiveness in predicting patient outcomes.

**Figure 4:**
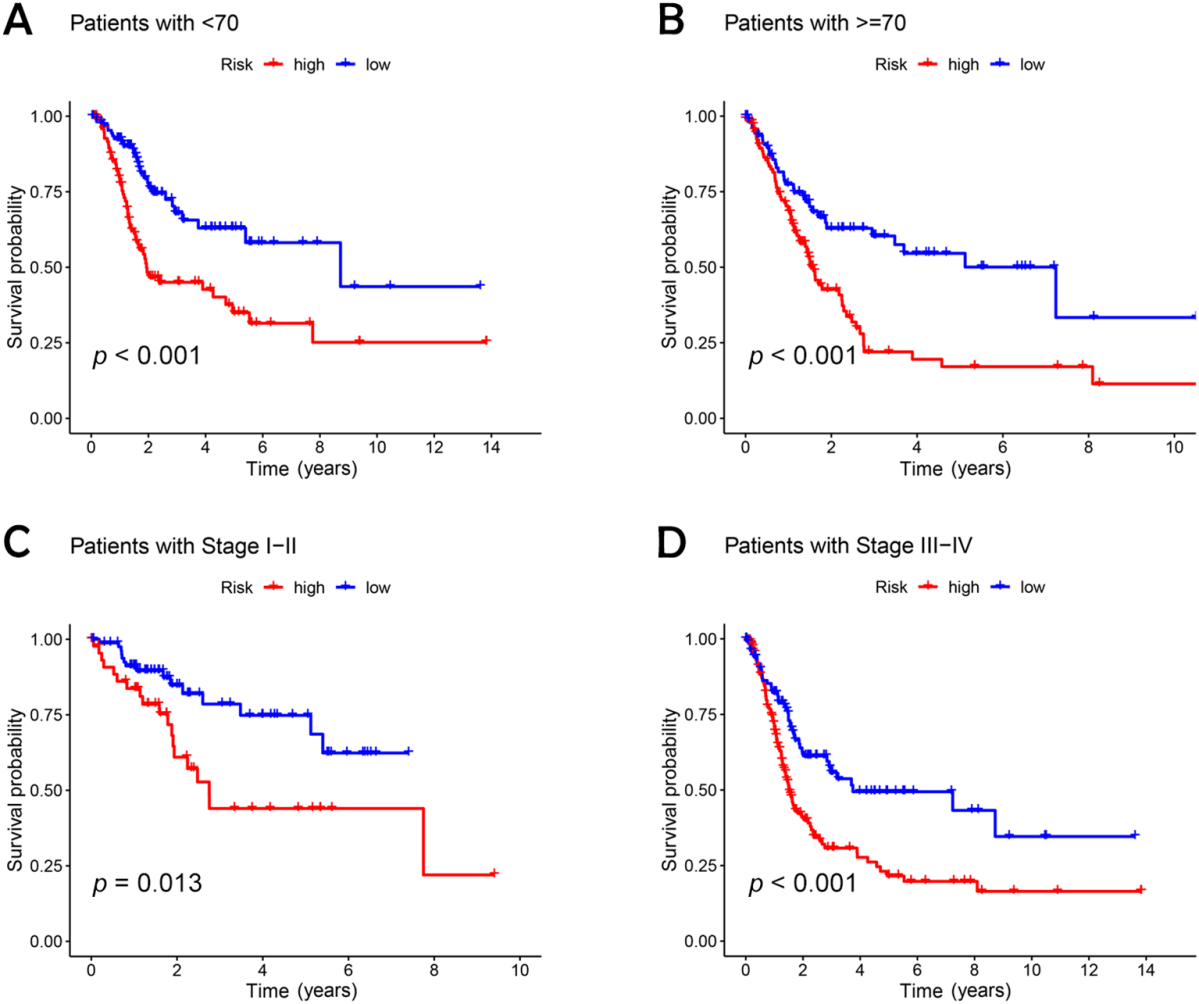
Kaplan-Meier survival analysis of high- and low-risk patients based on different clinical variables. **(A, B)** age; **(C, D)** stage.

**Figure 5:**
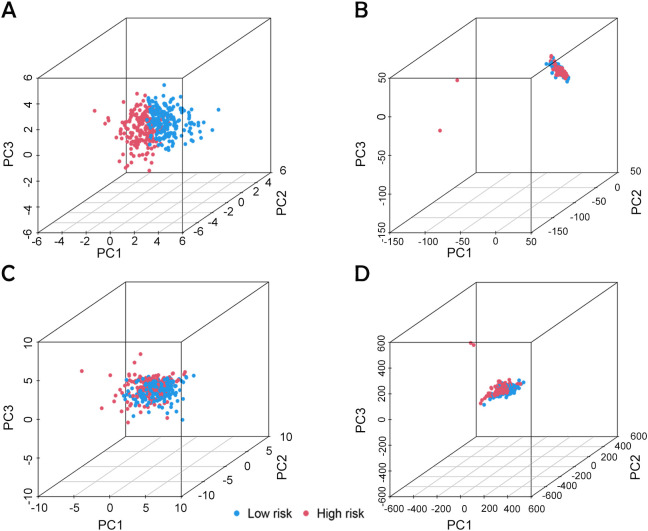
PCA analysis based on different classification indexes. **(A)** Model lncRNA, **(B)** Bisulfide-dead lncRNA, **(C)** Bisulfide-dead genes and **(D)** all genes.

An analysis of the heatmap showed elevated levels of *MIR4435-2HG* and *ARHGAP5-AS1* as biomarkers of poor prognosis in high-risk individuals. In contrast, *AC005840.4*, *AC010331.1*, and *AL021707.6* showed lower expression in those at high risk, suggesting their role as favorable prognostic indicators (**Figures 3G–I**). Moreover, analysis of progression-free survival revealed that individuals classified as low-risk enjoyed notably extended durations of high-quality survival. (*p* < 0.001; **Figure 3M**), which aligns with their superior OS relative to the high-risk cohort. Further analysis, accounting for clinical variables like age and cancer stage, consistently showed reduced OS in high-risk patients, irrespective of these factors (**Figures 4A–D**). Principal component analysis (PCA) demonstrated that the lncRNA-based model efficiently distinguished between patients with elevated and reduced risk, showing significantly better performance than other categorical indicators (**Figures 5A–D**). The analysis of the DRL-based prognostic model revealed an inverse relationship between DRL risk scores and OS in BC patients. Higher DRL risk scores were associated with shorter OS and poorer prognostic outcomes.

### 3.3 Development of a DRLs-based risk-prognostic model as a robust predictor of clinical outcomes in BC patients

**Figure 6:**
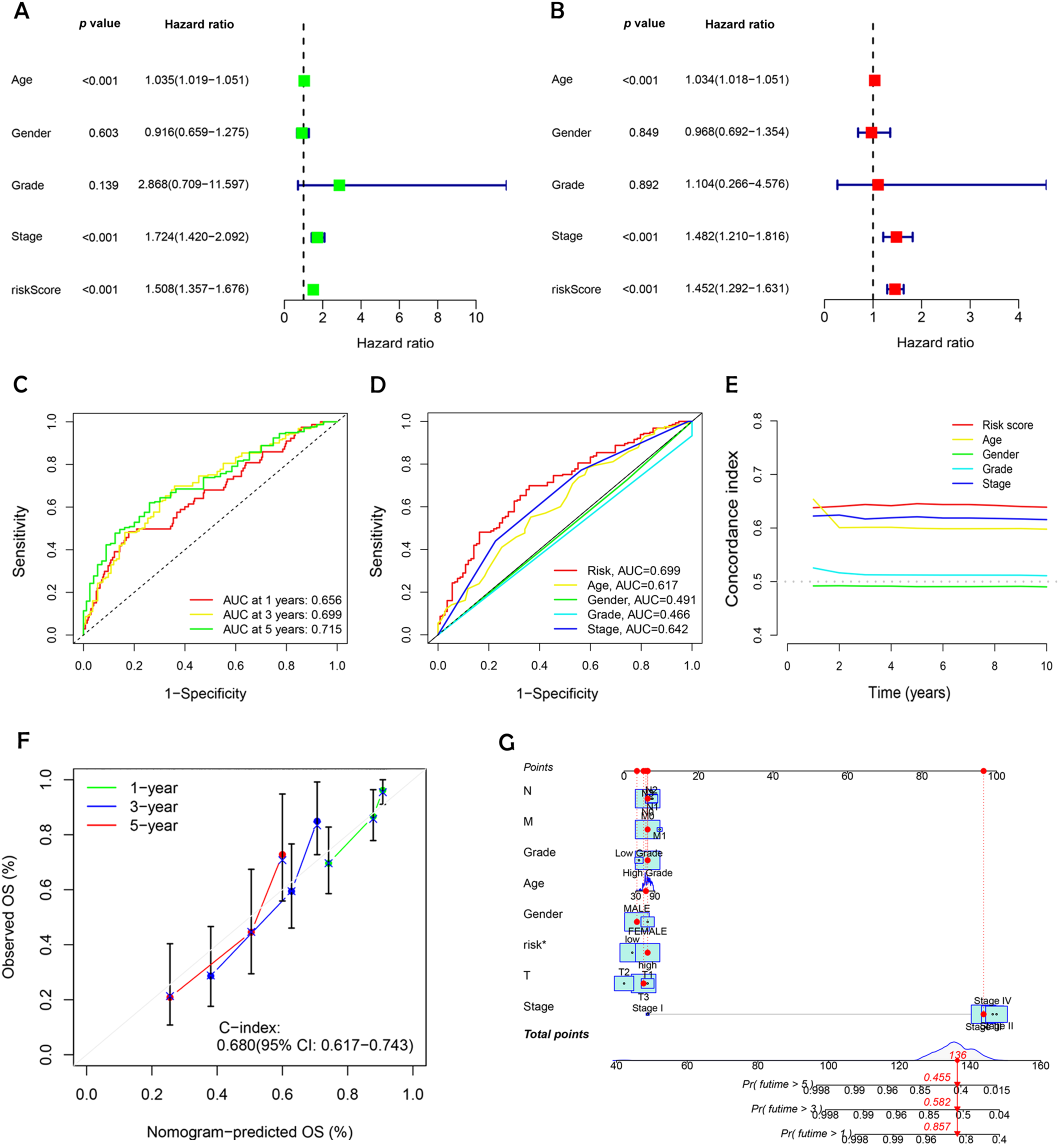
Evaluating the prognostic power and predictive accuracy of the disulfidptosis-related lncRNA risk scoring model. **(A)** Univariate Cox regression analysis evaluating clinical variables and risk scores. **(B)** Multivariate Cox regression analysis assessing clinical variables and risk scores. **(C)** Predicted 1-, 3-, and 5- year OS for BC patients using the risk scoring model. **(D)** Comparison of the risk scoring model with other clinical variables in predicting OS for BC patients. **(E)** Bar charts illustrating the predictive capability of risk scores and clinical variables for 1-, 3-, and 5-year OS in BC patients. **(F)** C-index ROC curves indicating the model’s consistency. **(G)** Calibration curves validating the model’s accuracy in predicting 1-, 3-, and 5- year OS in BC patients.

Univariate and multivariate Cox regression analyses were conducted to evaluate the prognostic relevance of clinical factors such as age, gender, cancer stage, and risk score. The risk score was found to be statistically significant (p < 0.001), confirming its independent predictive value for BC outcomes (**Figures 6A** and **6B**). To evaluate the model's effectiveness in predicting 1-, 3-, and 5-year OS, ROC curves and AUC values were employed (**Figure 6C**). The risk score achieved an AUC of 0.699 (**Figure 6D**), demonstrating greater predictive power than most clinical factors, except for staging. The C-index curve (**Figure 6E**) demonstrated that the concordance index of the risk score remained consistently higher than other clinical factors over time, further reinforcing the model's reliability. Calibration plots were generated to estimate OS by combining the risk score with clinical parameters. These plots indicated survival rates of 85.7%, 58.2%, and 45.5% at 1, 3, and 5 years, respectively (**Figure 6F**). The calibration curve (**Figure 6G**) supported the accuracy of these predictions. These findings confirm that the DRL-based risk model is a dependable predictor of survival outcomes in BC patients, operating independently of other clinical factors.

### 3.4 Pathway and functional enrichment insights through GO, KEGG, and GSEA in BC

**Figure 7:**
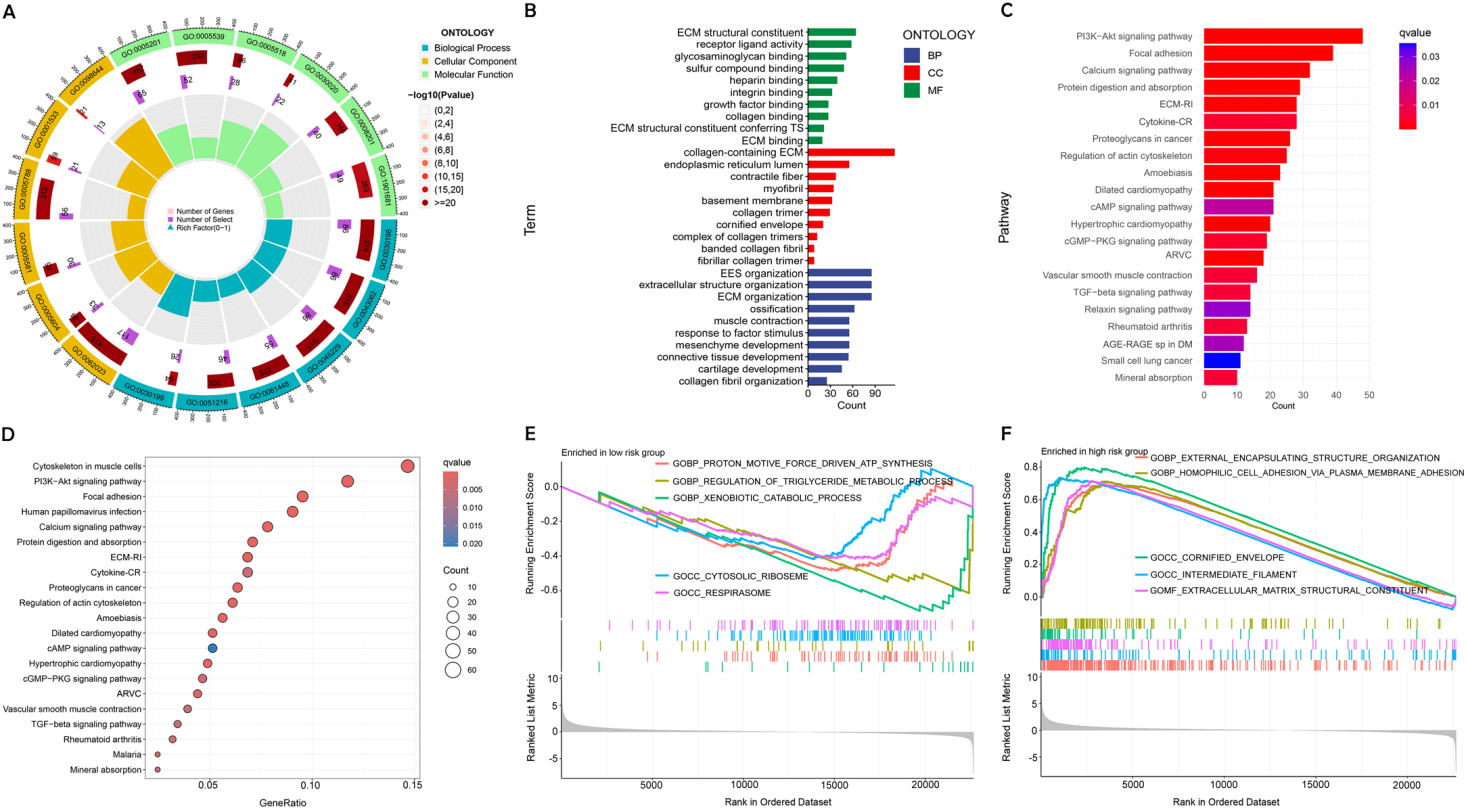
Enrichment analysis using GO, KEGG, and GSEA. **(A, B)** Diverse molecular BPs, CCs, and molecular functions are revealed through GO analysis. **(C, D)** Significant pathways are identified in the KEGG pathway analyses. **(E, F)** GSEA highlights the top five pathways enriched in high- and low-risk populations.

We performed GO and KEGG pathway analysis to deeply explore the molecular functions associated with the identified DRLs in BC. The GO enrichment analysis of DEGs revealed substantial enrichment in biological processes critical to immune response and extracellular matrix organization, highlighting their potential roles in BC progression. Cellular component analysis indicated significant involvement of DEGs in the extracellular matrix and endoplasmic reticulum, while molecular function analysis showed enrichment in protein binding and receptor activity, suggesting these interactions are vital for tumor development and immune regulation (**Figures 7A, B**).

DEGs were shown to be primarily involved in several crucial signaling pathways relevant to BC biology, as indicated by KEGG pathway enrichment analysis. These pathways are known to be crucial for processes such as cell survival and migration, underscoring their importance in the context of cancer progression (**Figures 7C, D**).

GSEA further identified distinct biological pathways activated in different risk categories. Pathways linked to cellular structure and adhesion showed significant enhancement in the group at high risk. indicating a potential link to the more aggressive cancer phenotype. Elevated metabolic activity was noted among low-risk patients, potentially explaining their better prognosis (**Figures 7E, F**).

### 3.5 Immune microenvironment differences in BC patients with varying risk levels

**Figure 8:**
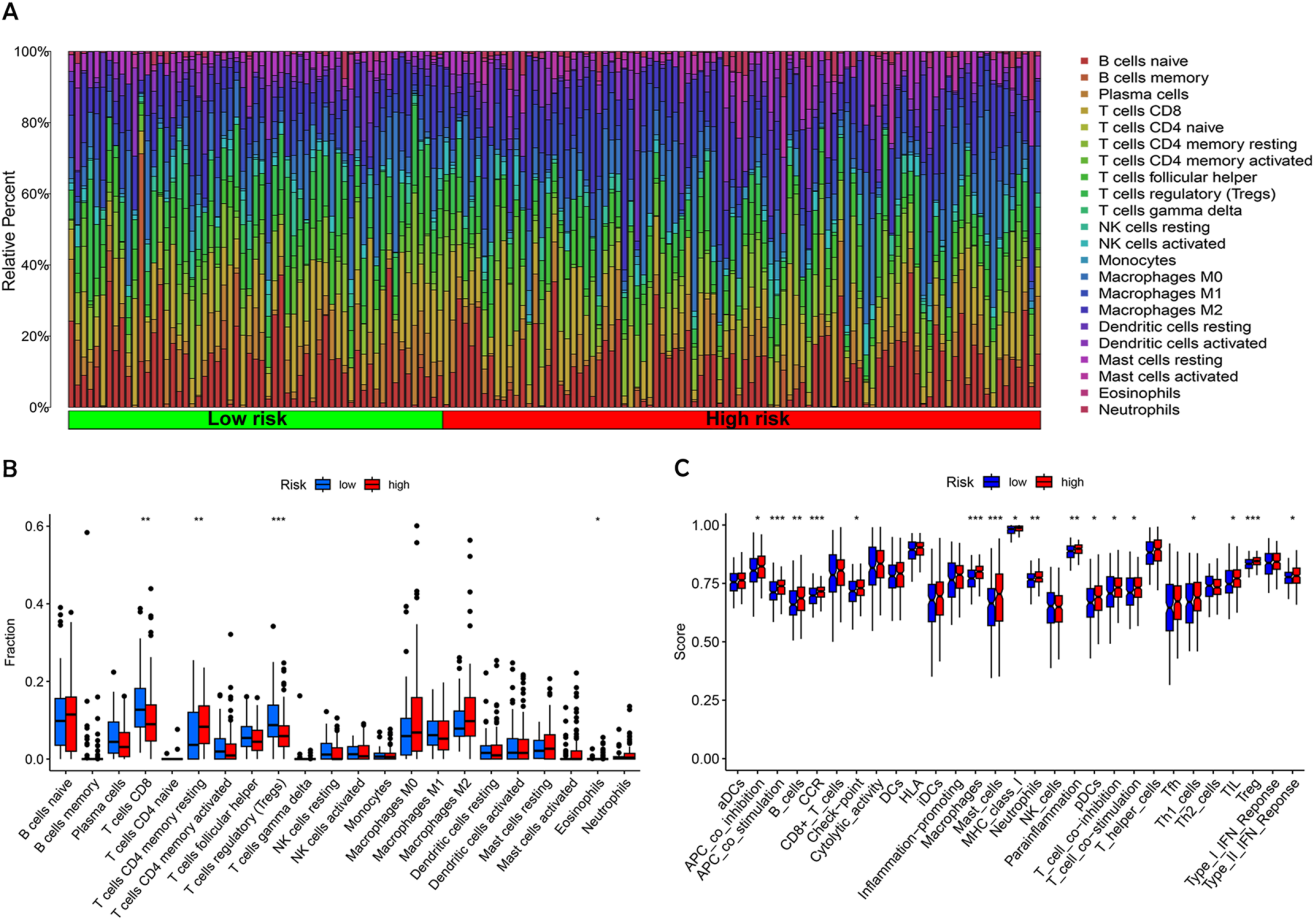
Analysis of tumor immune microenvironment in high-risk and low-risk patient groups. **(A)** The proportion of 22 different tumor-infiltrating immune cells. **(B)** Violin plot depicting the proportion of the 22 tumor-infiltrating immune cells from **(A)**. **(C)** Comparative analysis of immune-related functions between high-risk and low-risk groups. Statistical significance: *p < 0.05; **p < 0.01; ***p < 0.001; ns, no significance.

BC progression is inextricably linked to the tumor immune microenvironment. Immune cells play various roles within BC, either suppressing the immune response or stimulating anti-tumor immunity, significantly influencing the tumor growth process (19). We assessed immune cell infiltration in tumors across patient groups with varying risk levels (**Figure 8A**). The analysis indicated that CD8+ T cells and regulatory T cells (Tregs) were significantly less abundant in the high-risk group compared to the low-risk group. The levels of resting memory CD4+ T cells and eosinophils were found to be lower in patients categorized as low-risk compared to those in the high-risk group (**Figure 8B**).

To delve deeper into the immune landscape, Gene Set Variation Analysis (GSVA) was employed to assess variations in pathway-related gene sets among samples. This analysis highlighted that genes associated with APC co-stimulation, CCR, mast cells, macrophages, and Tregs were notably enriched in the high-risk group (**Figure 8C**). These findings underscore a notable variation within the immune landscape of tumors across different risk levels. The low-risk group, benefiting from a stronger immunosurveillance effect, showed higher levels of resting immune cells, particularly resting memory CD4+ T cells. In contrast, immune cells associated with tumor invasion, metastasis, and BC progression, such as T cells and NK cells, for instance, were more prevalent in individuals at greater risk.

### 3.6 TMB analysis and survival analysis of TMB

**Figure 9:**
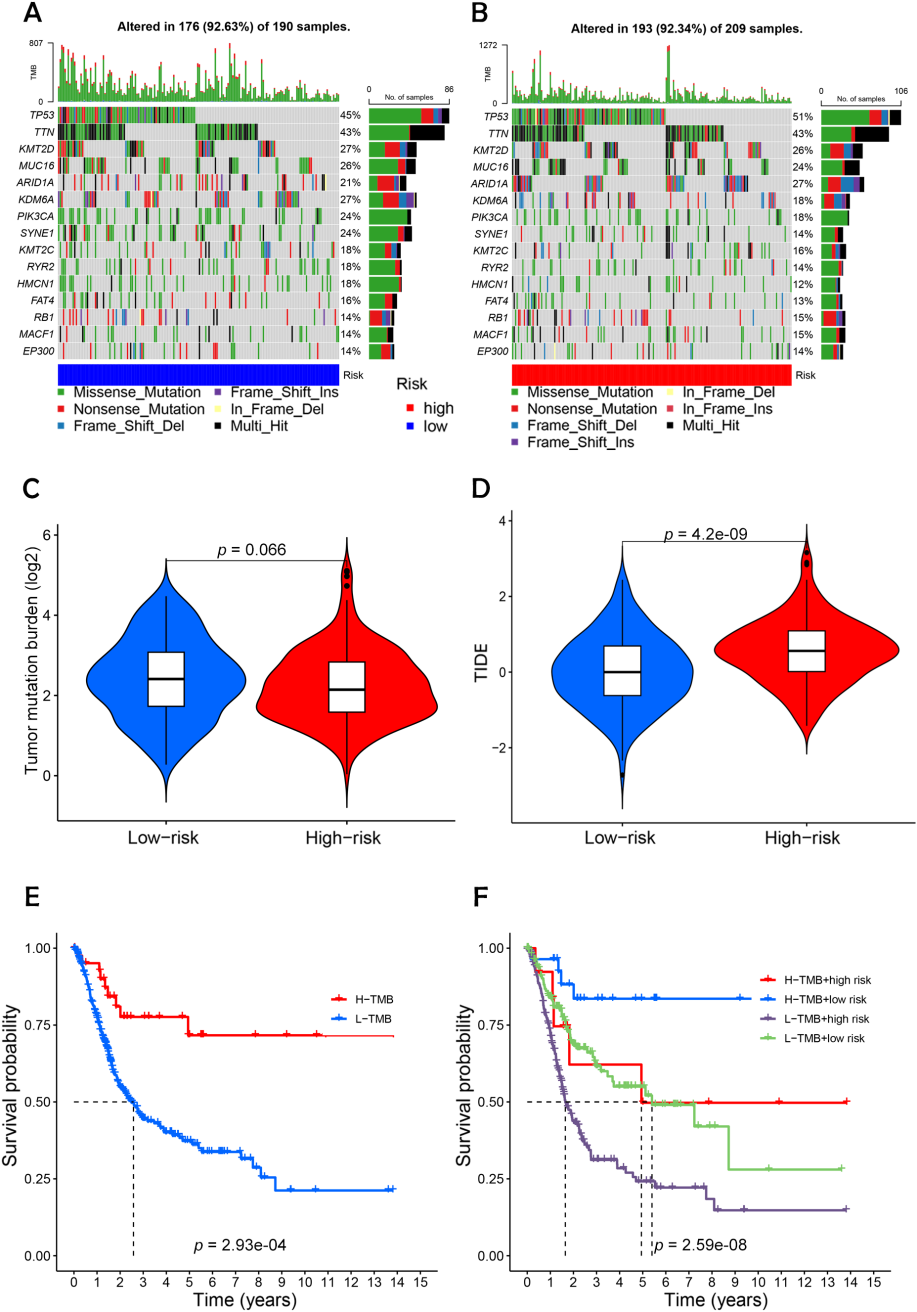
Differential analysis of tumor mutation burden with tumor immune dysfunction and exclusion. **(A, B)** Waterfall plots depicting 15 highly mutated genes in the high- and low-risk node BC groups. **(C)** Differential analysis of TMB in patients in the high- and low-risk BC groups. **(D)** Analysis of TIDE of patients in the high- and low-risk groups. **(E)** TMB survival curves of high-mutated and low-mutated groups. **(F)** TMB survival curves for different high and low risk groups versus high and low mutation groups. Statistical significance: *p* < 0.05; *p* < 0.01; *p* < 0.001; ns, no significance.

Using the maftools package in R, somatic mutation data from the TCGA database were analyzed to create waterfall plots, illustrating genetic alterations in high-risk versus low-risk BC groups (**Figures 9A, B**). This analysis highlighted 15 genes with notable mutation frequencies. In 209 patients classified as high-risk, mutations were present in 92.34% of cases, with genes such as TP53, TTN, and ARID1A showing more frequent mutations compared to the low-risk group. Conversely, genes like KDM6A, SYNE1, MUC16, and PIK3CA were more commonly mutated in the low-risk group. Further analysis of TMB and TIDE scores indicated a higher immune escape potential in the high-risk group (**Figures 9C, D**), although TMB differences between the groups were not statistically significant (p = 0.066). Survival probability analysis revealed that patients in the high-TMB, low-risk group had the highest survival rates, while those in the low-TMB, high-risk group exhibited the lowest survival rates (**Figures 9E, F**). This suggests that high mutational load combined with low-risk status may correlate with better survival, potentially due to greater immune diversity and stronger immune activation signals in these tumors.

Furthermore, the analysis of TMB indicated a higher mutational load in the high-risk group, though these findings were not statistically significant (20). The concept of TIDE, which measures the capacity of tumor cells to evade immune detection and suppress immune responses, showed that revealed that the high-risk cohort demonstrated notably elevated TIDE scores compared to the low-risk cohort.

Survival analyses demonstrated that the group with high mutations had a better prognosis than those with fewer mutations, particularly noting that the high-mutation, low-risk group had the highest survival probability. This suggests that these tumor cells might possess greater immune diversity and stronger immune activation signals. The data indicate a prevalent strategy of immune evasion and frequent mutations in tumor cells of low-risk BC patients, highlighting the substantial therapeutic promise of immune checkpoint inhibitors for treating low-risk BC.

### 3.7 Assessment of drug sensitivity to BC

**Figure 10:**
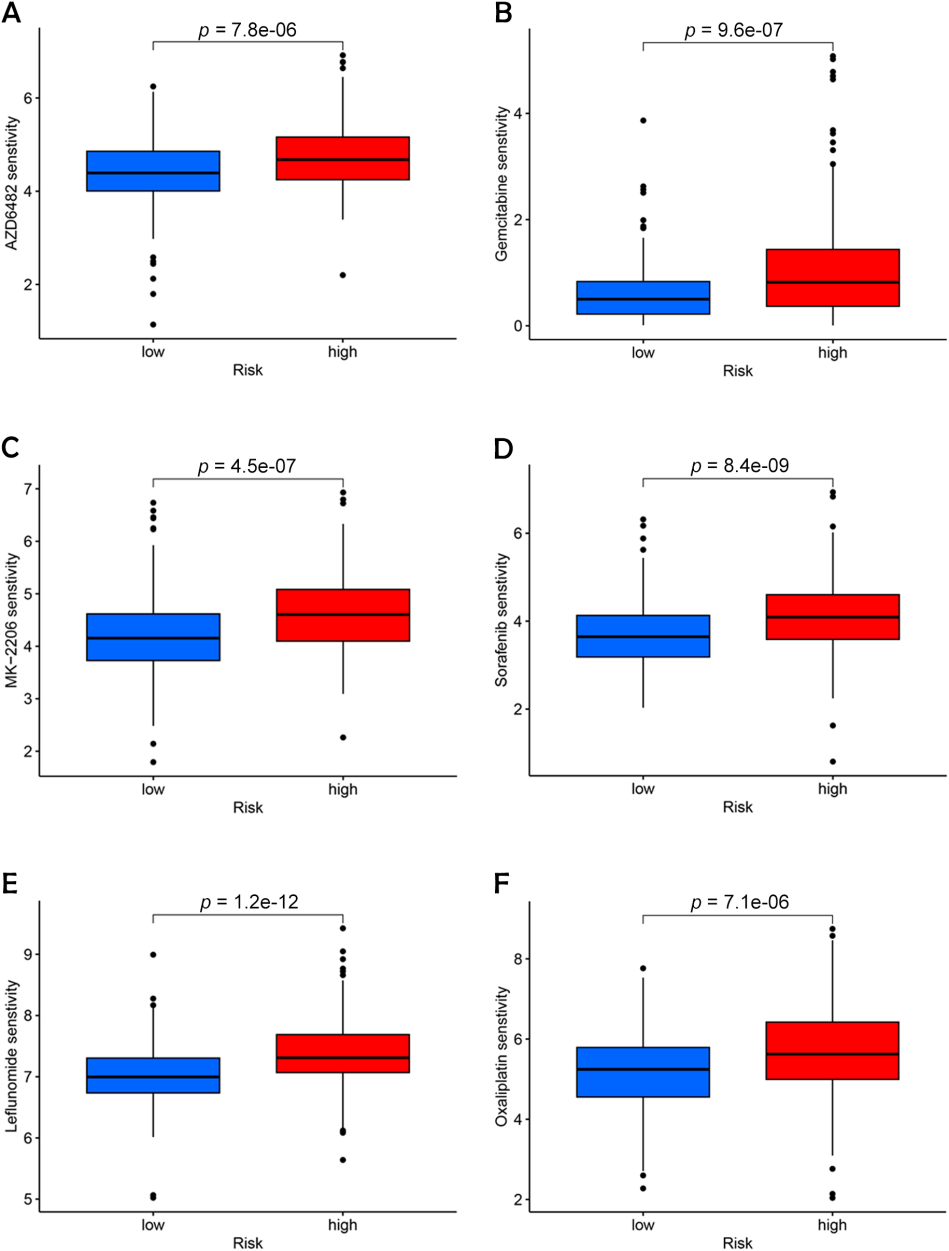
Identification of potential drugs for the treatment of BC. **(A)** AZD6482. **(B)** Gemcitabine. **(C)** MK-2206. **(D)** Sorafenib. **(E)** Leflunomide. **(F)** Oxaliplatin.

Sensitivity analysis revealed that high-risk patients exhibited significantly higher sensitivity to all six drugs compared to low-risk patients (**Figures 10A–F**). This outcome suggests that targeting these pathways may support personalized treatment strategies tailored for high-risk BC patients (21).

The role of chemotherapy in enhancing the prognosis of patients with LSCC is well-established. In this study, we employed OncoPredict to evaluate the efficacy of 21 different drugs and identified six that were most representative and clinically significant: *Sorafenib*, *Oxaliplatin*, *MK-2206*, *Gemcitabine*, *AZD6482*, and *Leflunomide*. Among these, *Sorafenib*, a well-known anti-angiogenic agent, has demonstrated broad-spectrum efficacy across various cancer types by inhibiting angiogenesis, which in turn reduces tumor growth and induces apoptosis and necrosis (22).

*Oxaliplatin* has emerged as a valuable alternative to cisplatin, particularly in the chemotherapy of advanced BC, due to its reduced nephrotoxicity, making it a safer option for long-term treatment regimens (23). *MK-2206*, an AKT inhibitor that specifically targets the PI3K-AKT-mTOR pathway, has shown promising therapeutic potential in the management of urothelial BCs, offering a targeted approach to disrupting cancer cell survival pathways (24). *Gemcitabine*, another critical agent, serves as a key alternative to Bacillus Calmette-Guerin (BCG) for patients with high-risk non-muscle-invasive BC, especially during times of BCG scarcity, where its availability is limited. This drug’s efficacy in such contexts underscores its importance in maintaining continuity of care for these patients (25). *AZD6482*, a novel isoform-selective PI3Kβ inhibitor, has been identified as a potential therapeutic target due to its ability to disrupt the enzyme’s interaction with ATP, with studies confirming its effectiveness in sensitizing BC cells, thus providing a new avenue for targeted therapy (26). Moreover, *Leflunomide*, recognized for its immunomodulatory effects, has been shown to greatly reduce the viability of cells in BC by inhibiting a key signaling pathway (27).

### 3.8 Induction of disulfidptosis and apoptotic cell models and *in vitro* validation of risk prediction models

**Figure 11:**
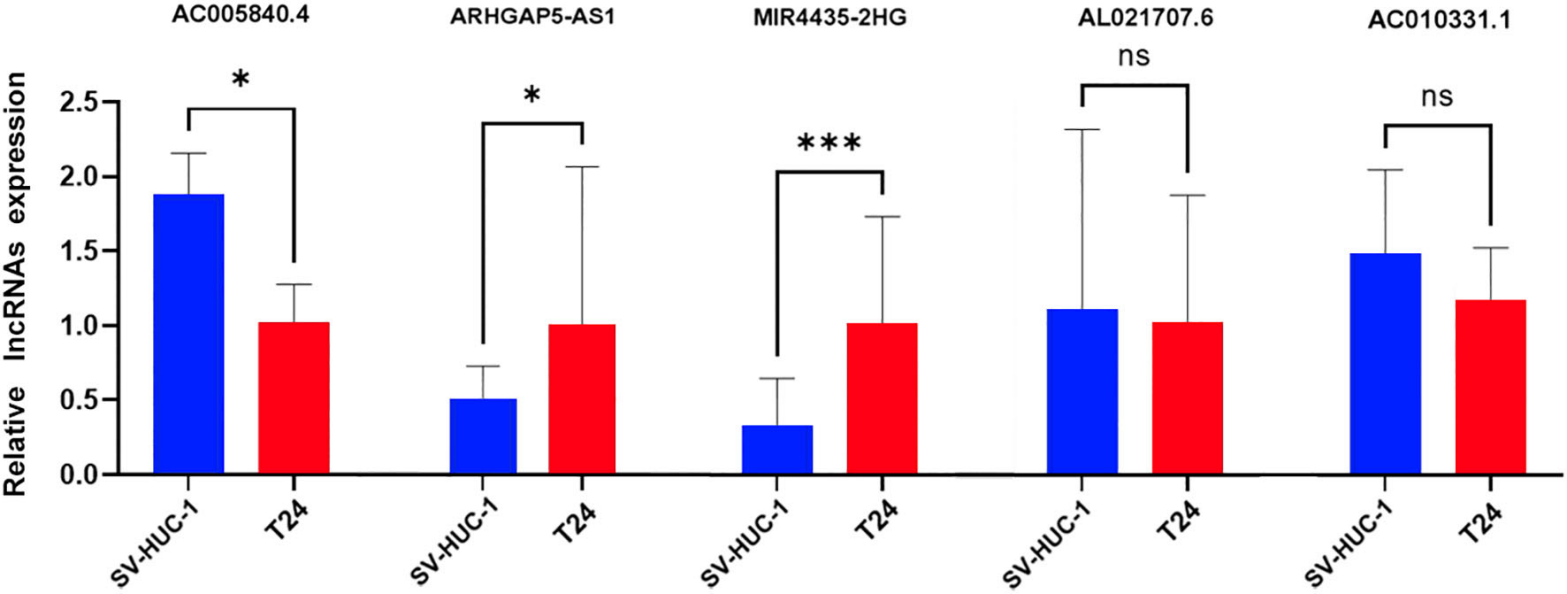
Validation of 5-DRLs expression in cell lines. The expression of lncRNAs in SV-HUC-1 and T24 cells was measured by quantitative real-time polymerase chain reaction (n = 3). Statistical significance: *p < 0.05; ***p < 0.001; ns, no significance.

To confirm the expression patterns of the five DRLs identified in our study, we conducted RT-qPCR on BC cell lines. As shown in **Figure 11**, the expression levels of *MIR4435-2HG* and *ARHGAP5-AS1* were significantly elevated in BC cells (T24) compared to normal human bladder cells (SV-HUC-1). In contrast, *AC005840.4* exhibited significantly lower expression in BC cells, while *AC010331.1* and *AL021707.6* exhibited no notable differences in expression between cancerous and normal cell types.

## 4 Discussion

Cell death, a fundamental physiological process, is vital for normal development and the maintenance of homeostasis within organisms. It has become a central focus in oncology because manipulating the pathways that regulate cell death can selectively target and destroy cancer cells, thereby offering a promising avenue for therapeutic interventions (14). One of the latest breakthroughs in this field is the identification and comprehensive characterization of disulfidptosis, a unique mechanism of cell death. This discovery has not only broadened our understanding of cellular demise but has also opened up novel avenues for the development of innovative cancer therapies that could be more effective against resistant cancer types. In this context, GLUT1 inhibitors, such as WZB117, represent a promising tool for potentially inducing disulfidptosis in cancer cells overexpressing SLC7A11, given their dependence on glucose uptake. Although this study did not employ a GLUT1 inhibitor experimentally, this theoretical application may serve as a direction for future studies, particularly for developing targeted therapies in BC treatment.

However, despite these significant advancements, the role of disulfidptosis in the specific context of BC remains largely uncharted. There is a need for in-depth exploration to fully understand how this mechanism might influence the pathophysiology of BC and its potential as a therapeutic target. To address this gap, our study was meticulously designed to investigate the importance of DRLs in BC through a series of comprehensive correlation analyses. Our research highlights the intricate and multifaceted role of disulfidptosis in the onset and progression of BC by examining these specific molecular interactions (12, 28, 29). In this investigation, we created a predictive risk model that incorporates five key DRLs. This model enables the classification of patients into separate risk categories, supporting more tailored treatment strategies. The reliability and autonomous predictive ability of this model were thoroughly validated through an array of advanced analytical methods, such as ROC curve evaluation, C-index analysis, calibration plots, as well as univariate and multivariate Cox regressions (30). These methods collectively ensured the reliability of our findings.

Following the application of this risk model, we conducted a comparative analysis of survival outcomes between the defined risk groups. The results clearly demonstrated a positive association between elevated risk scores and higher mortality rates in patients with BC (28, 31). These findings highlight the value of the risk score as an accurate and dependable method for forecasting patient survival. which could be instrumental in guiding clinical decision-making and optimizing treatment strategies.

To bolster the reliability of our risk model, we conducted an analysis of mRNA expression for five DRLs in two different cell lines, T24 and SV-HUC-1. To confirm the specificity of lncRNA expression, melting curve analyses were conducted, and the results are provided in the supplementary materials, validating primer specificity across experimental runs. Additionally, we performed enrichment analyses on DEGs between high-risk and low-risk patient groups, finding that these DEGs were primarily involved in immune-related processes. Additionally, DRLs were significantly associated with various biological pathways related to immune responses and cellular signaling. Gene set enrichment analyses further indicated elevated pathway activity within the high-risk group.

Neutrophils release chromatin DNA strands surrounded by granular proteins, forming NETs to capture microbes. Research has underscored the role of NET formation in the development and progression of cancer. Neuroendocrine tumors are known for enhancing vascular permeability, which aids the spread of malignant cells from the bloodstream to remote sites. NET-DNA, via its transmembrane receptor CCDC25, can act as a chemotactic agent for cancer cells, promoting the mobility of tumor cells by triggering the ILK-β-parvin signaling cascade. The IL-17 signaling pathway is implicated in cancer development and is closely associated with inflammation progression. Adenomatous intestinal epithelial cells in mice carrying the Apc mutation undergo rapid proliferation when stimulated by IL-17 signaling, promoting adenoma formation (32). Adenomas impair the intestinal barrier function and amplify the IL-17 response within tumors, thereby accelerating the growth of the tumor. The nuclear receptor PPAR, once activated by its ligand, plays a role in regulating energy balance and lipid metabolism. Aberrant activation of the PPAR signaling system has been observed in BC tumor cells (33). When the pathway was blocked with PPARγ inhibitors, tumor epithelial cell proliferation was significantly inhibited, and apoptosis increased.

In our analysis, the low-risk cohort presented higher TMB and lower TIDE scores, indicating a greater likelihood of response to immune checkpoint inhibitors and reduced immune evasion potential. In contrast, individuals at greater risk displayed heightened indicators associated with a more suppressive tumor microenvironment. Further studies are necessary to validate the efficacy of these checkpoint inhibitors, particularly in breast cancer, where certain pathway alterations might enhance responsiveness to immunotherapies. Additionally, IFNG plays a significant role in tumor immune angiogenesis (34).

This research provides insight into the foundational mechanisms of disulfidptosis in BC, underscoring its potential as a prognostic marker. Through the evaluation of gene expression variations between the different risk groups, we identified critical pathways influencing immune response and disease outcomes in BC (35). These findings offer substantial implications for advancing both preventive and therapeutic strategies, positioning this study as a leading effort in the intersection of disulfidptosis, lncRNAs, and immunotherapy in BC research.

Despite these findings, our research had certain intrinsic limitations. As our study relied solely on data from the TCGA database for both model training and validation, this may limit the generalizability of our findings. To enhance the robustness and broader applicability of our model, future research should incorporate external cohorts across diverse patient populations. Additionally, experimental validation of DRLs in functional studies will be essential for a more comprehensive understanding of their biological roles in BC pathophysiology. Expanding future research to include *in vivo* and *in vitro* experiments will help clarify the fundamental mechanisms underlying DRLs and further establish their potential as therapeutic targets.

## 5 Conclusions

Overall, our study identified five key disulfidptosis-related lncRNAs (*AC005840.4*, *AC010331.1*, *AL021707.6*, *MIR4435-2HG* and *ARHGAP5-AS1*) and developed a prognostic model with high accuracy in predicting survival rates for BC patients. These findings provide a foundation for future research to develop precision treatment strategies targeting these molecular markers, potentially improving clinical outcomes and personalized patient management.

## Data Availability

The original contributions presented in the study are included in the article/**Supplementary Material**. Further inquiries can be directed to the corresponding authors.
