# Supplementary material for: Prognostic model based on disulfidptosis-related lncRNAs for predicting survival and therapeutic response in bladder cancer
Source: Front Immunol. 2024 Dec 2;15:1512203. doi: 10.3389/fimmu.2024.1512203 (PMC11647029; doi:10.3389/fimmu.2024.1512203)
Supplement: Supplementary file 1 [file DataSheet1.docx]

Supplementary Material

| **Gene Name** | **Forward Primer** | **Reverse Primer** |
| --- | --- | --- |
| MIR4435-2HG | 5‘-ATGACTGGATGGTCGCTGCTT-3’ | 5‘-TCCCAGGAACTGTGCTGTGAA-3’ |
| ARHGAP5-AS1 | 5‘-CACAAGTTTTGGGGTAGAGCA-3’ | 5‘-GCAAGGCAGATTCCGATGA-3’ |
| AC010331.1 | 5‘-GGAGTCGAGGATGAAGGAAAT-3’ | 5‘-GAGACCAGCTAGGGTGAGTGAT-3’ |
| AL021707.6 | 5‘-CGGGGAGAAGGTCTGAGTG-3’ | 5‘-CTGTAGGAGGCGGGGAAG-3’ |
| AC005840.4 | 5‘-GGAGGGACTCAGGGGAGGT-3’ | 5‘-TGGAAGAAGCAAAAGCAGAAAC-3’ |

**Supplementary Table S1. Primer sequences for the samples used for qPCR.**

| Covariates | Type | Total | Test | Train | *p* value |
| --- | --- | --- | --- | --- | --- |
| Age | $\leq$65 | 159 (39.36%) | 75 (37.13%) | 84 (41.58%) | 0.4152 |
| Age | > 65 | 245 (60.64%) | 127 (62.87%) | 118 (58.42%) |  |
| Gender | Female | 106 (26.24%) | 55 (27.23%) | 51 (25.25%) | 0.7344 |
| Gender | Male | 298 (73.76%) | 147 (72.77%) | 151 (74.75%) |  |
| Grade | High Grade | 381 (94.31%) | 194 (96.04%) | 187 (92.57%) | 0.2467 |
| Grade | Low Grade | 20 (4.95%) | 7 (3.47%) | 13 (6.44%) |  |
| Grade | unknow | 3 (0.74%) | 1 (0.5%) | 2 (0.99%) |  |
| Stage | Stage I | 2 (0.5%) | 1 (0.5%) | 1 (0.5%) | 0.6743 |
| Stage | Stage II | 128 (31.68%) | 63 (31.19%) | 65 (32.18%) |  |
| Stage | Stage III | 140 (34.65%) | 76 (37.62%) | 64 (31.68%) |  |
| Stage | Stage IV | 132 (32.67%) | 62 (30.69%) | 70 (34.65%) |  |
| Stage | unknow | 2 (0.5%) | 0 (0%) | 2 (0.99%) |  |
| T | T0 | 1 (0.25%) | 1 (0.5%) | 0 (0%) | 0.7133 |
| T | T1 | 3 (0.74%) | 2 (0.99%) | 1 (0.5%) |  |
| T | T2 | 117 (28.96%) | 55 (27.23%) | 62 (30.69%) |  |
| T | T3 | 193 (47.77%) | 101 (50%) | 92 (45.54%) |  |
| T | T4 | 57 (14.11%) | 29 (14.36%) | 28 (13.86%) |  |
| T | unknow | 33 (8.17%) | 14 (6.93%) | 19 (9.41%) |  |

**Supplementary Table S2. Clinical characteristics of colorectal cancer patients in the training and test groups, n (%).**


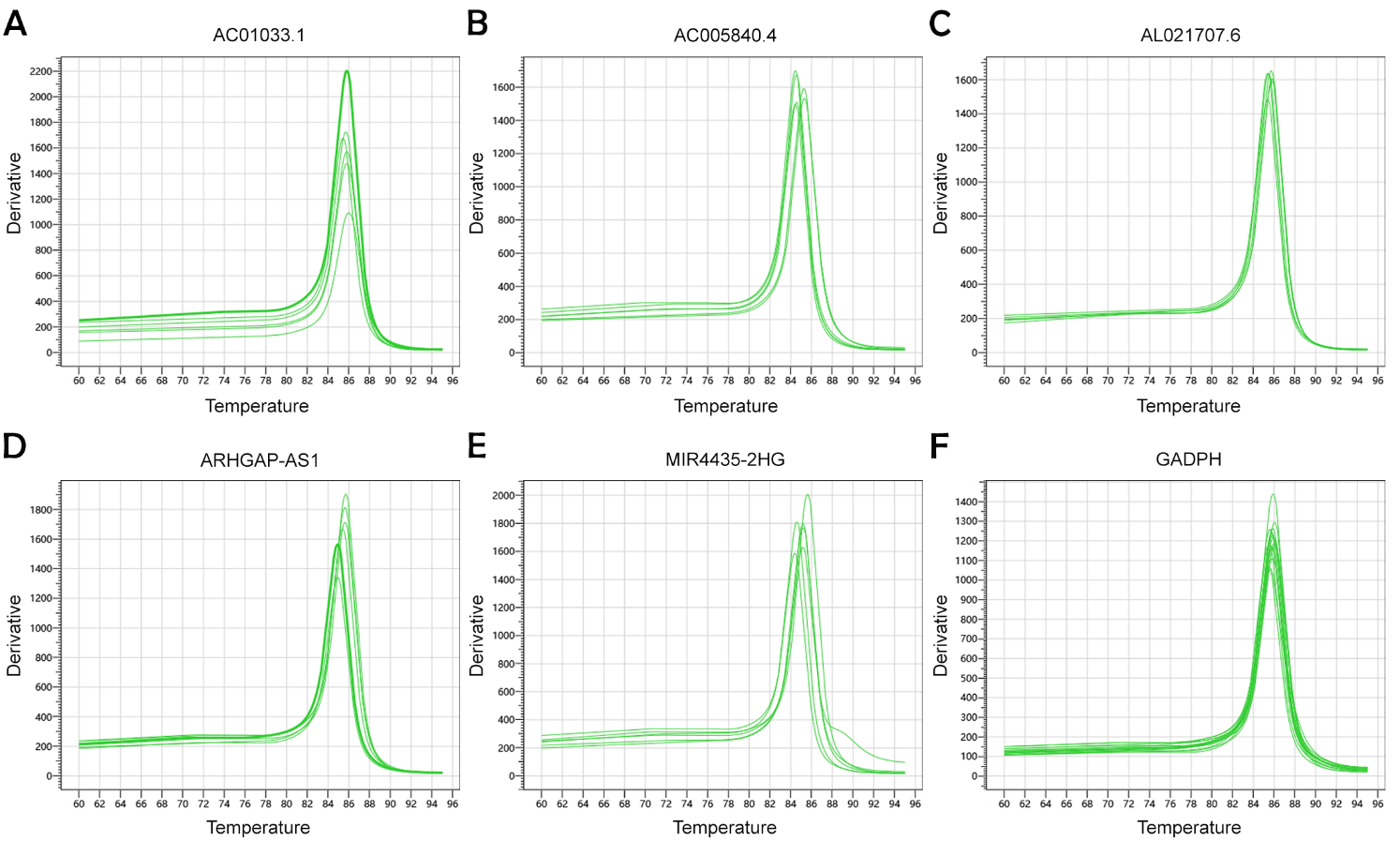


**Supplementary Figure S1. Melting curves for qPCR analysis of disulfidptosis-related lncRNA expression in T24 and SV-HUC-1 cells.**
